# Tobacco smoking associates with *NF1* mutations exacerbating survival outcomes in gliomas

**DOI:** 10.1186/s40364-022-00430-z

**Published:** 2022-11-09

**Authors:** Xi Li, Han Yan, Jun Wu, Longbo Zhang

**Affiliations:** 1 Institute of Clinical Pharmacology, Hunan Key Laboratory of Pharmacogenetics, Central South University, 410008 Changsha, China; 2 National Clinical Research Center for Geriatric Disorders (Xiangya Hospital), Central South University, Changsha, 410008 China; 3 Department of Pharmacy, The Second Xiangya Hospital, Central South University, 410011 Changsha, China; 4 Departments of Neurosurgery, Xiangya Hospital, Central South University, 410008 Changsha, China; 5 Departments of Neurosurgery, and Cellular & Molecular Physiology, Yale School of Medicine, LH 403, 333 Cedar Street, CT 06520-8082 New Haven, USA

**Keywords:** Tobacco smoking, Glioma, *NF1* gene

## Abstract

Tobacco smoking is associated with increased risks of nearly 20 types of cancer. Although the association between smoking and gliomas, the most prevalent type of adult brain tumor, is still unconclusive, here, we found that the frequency of *NF1* mutations was significantly increased in the glioma patients with smoking history compared to non-smoking patients (24% vs. 10%, *P* = 0.021). *NF1* acts as a tumor suppressor gene is highly mutated in gliomas. The TCGA data analysis indicated that glioma patients carrying *NF1* somatic mutations have worse overall survival (median survival time: smoking 19.9 months vs. non-smoking 36.8 month; *P* = 0.0018). In addition, we revealed that the *NF1* and *IDH1* mutations were mutually exclusive suggesting *NF1* mutation has independent molecular mechanism involved in glioma biology.

**Supplementary Information:**

The online version contains supplementary material available at 10.1186/s40364-022-00430-z.

To the editor:

Tobacco smoking is one of the largest health risks worldwide which is associated with the continuum of human cancers leading more than 6 million deaths every year. Tobacco smoke is thought to cause DNA damage by DNA adducts, the bonding of reactive species of the carcinogen to DNA bases, which increases the burden of somatic mutations and ultimately elevates the chances of acquiring cancerogenesis driver mutations. This study aimed to identify the somatic alterations associated with the smoking history in individuals with gliomas.

We performed a targeted sequencing of a cohort of 184 gliomas. Five spatially distinct regions of tumor were heterogenized for tissue DNA extraction. Survival analyses were performed using 739 patients from The Cancer Genome Atlas (TCGA). Of 184 gliomas, 55 (29.89%) were from tobacco smokers and 129 (70.11%) from never-smokers with similar distribution of age, sex and pathological grade. Genomic profiling revealed that the median somatic tumor mutational burdens (TMB) of glioma DNA were not significantly different between smoking and non-smoking cohorts. The mutational landscapes in both cohorts were similar, where the top significantly mutated genes were *TP53, IDH, ATRX, PIK3CA, PTEN* and *NF1*, which aligned with previous findings from TCGA (Fig. 1 A). However, the frequency of *NF1* mutation is significantly increased in the smoking cohort compared to non-smoking cohort (24% vs. 10%, *P* = 0.021, OR: 2.745, 95% CI:1.077–7.016; Fig. 1B). *NF1* acts as a tumor suppressor gene coding neurofibromin which is a member of Ras-GTPase-activating protein-related proteins negatively regulating RAS/MEK pathway. Large-scale cancer genomics have indicated that *NF1* gene is highly mutated in gliomas. The development of genetically engineered mouse models by introducing *NF1* mutations further indicates that *NF1* gene alterations are determinative in tumor initiation and progression [1, 2]. Further assessment of the mutational spectrum indicated that the distributions of *NF1* alterations were similar in both smoking and non-smoking cohorts without significantly clustering into specific domains (Fig. 1 C). In addition, somatic interaction analysis suggested that *NF1* mutations exhibit mutual exclusivity from IDH1 mutations (Fig. 1D-E). To evaluate the effect of *NF1* mutation on prognoses of gliomas, we incorporated 739-case data from TCGA revealing that *NF1* somatic mutation is strongly associated with worse overall survival (five-year survival rate: smoking 9.0% vs. non-smoking 40.3%; median survival time: smoking 19.9 months vs. non-smoking 36.8 month; *P* = 0.0018, HR = 1.899, 95% CI: 1.104–3.265; Fig. 1 F). Together, we found that tobacco smoking is significantly associated with high frequency of *NF1* gene alterations leading poor overall survival in gliomas.

**Fig. 1:**
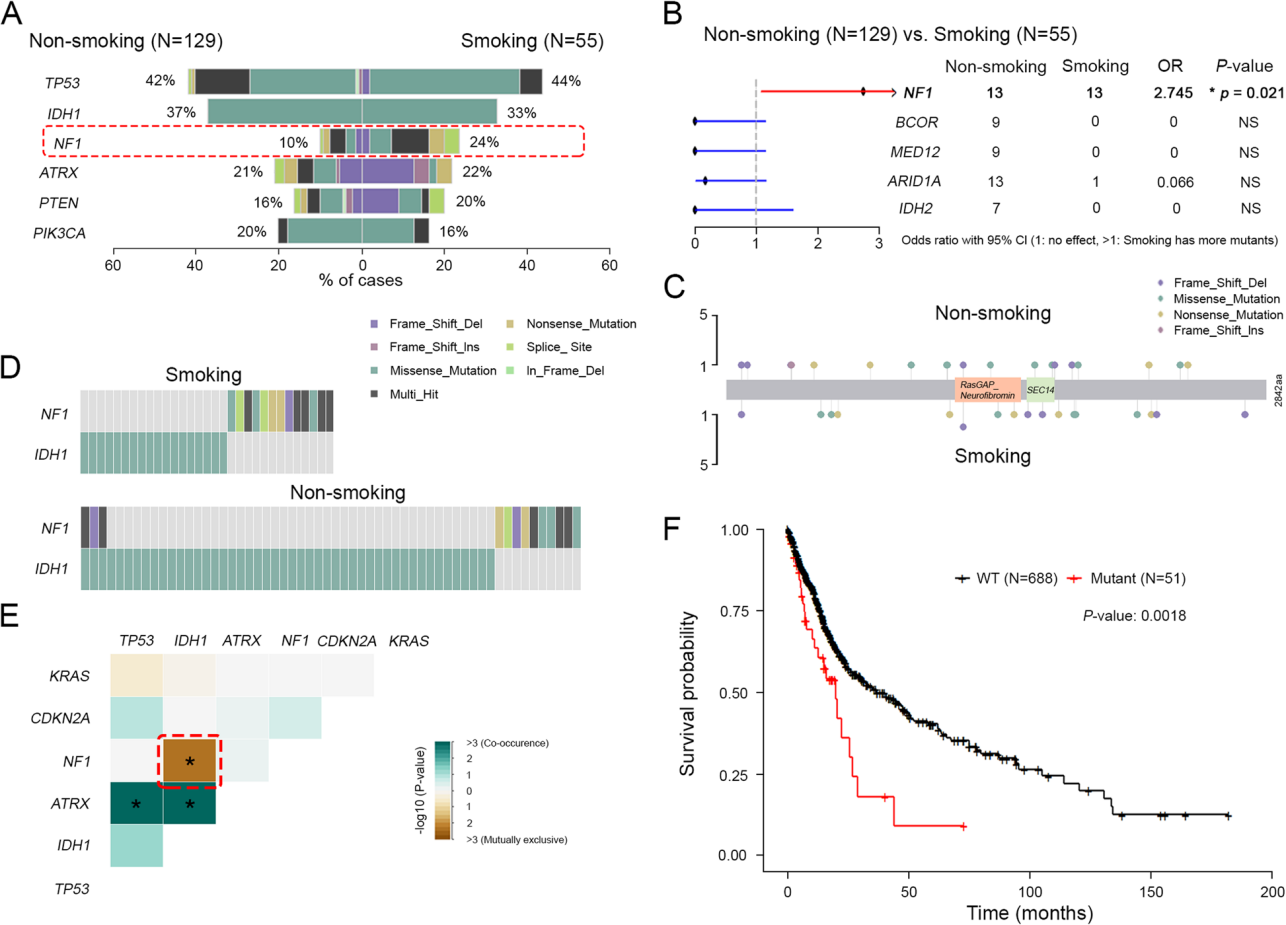
Tobacco smoking associates increased ***NF1*** mutation in gliomas. **A** Overview of mutant genes in smoking and non-smoking cohorts. **B** Forest plot of frequency of gene mutation. The frequency of *NF1* mutation is significantly higher in smoking group, *: *P* ˂ 0.05. **C** The spectrum of *NF1* variants is represented with each mutation shown only once per patient. **D-E** Correlation between gene mutations. *NF1* and *IDH1* mutations are mutually exclusive, *: *P* ˂ 0.05. **F** Survival analysis shows glioma patients with *NF1* mutations have worse overall survival. *P* = 0.0018

## Conclusion

Tobacco smoking increases cancer risk by increasing the somatic mutation loads in the tissues directly or indirectly exposed to smoke [3]. Although the association between smoking and gliomas remains uncertain [4–6], our data indicated that the frequency of *NF1* mutation is significantly elevated in the glioma patients with smoking history who presented worse overall survival. In addition, we revealed that the *NF1* and *IDH1* mutations were mutually exclusive suggesting *NF1* mutation has independent molecular mechanism involved in glioma biology, and implicating potential targeted therapies for this subgroup (*NF1* mutant, *IDH1* wildtype) of gliomas in which temozolomide resistance has been observed. MEK inhibitors such as selumetinib and trametinib are currently employed in several clinical trials for gliomas with *NF1* mutations which would potentially benefit clinical treatment of gliomas. The limitations of this study include the absence of mechanism exploration of how tobacco smoke leads the increased frequency of *NF1* mutation.

## Supplementary Information

**Additional file 1.**

## Data Availability

Data that support the findings of this study are available from the corresponding authors upon reasonable request.

## References

[1] Zhu Y, Guignard F, Zhao D, Liu L, Burns DK, Mason RP, Messing A, Parada LF (2005). Early inactivation of p53 tumor suppressor gene cooperating with NF1 loss induces malignant astrocytoma. Cancer Cell.

[2] Zuckermann M, Hovestadt V, Knobbe-Thomsen CB, Zapatka M, Northcott PA, Schramm K, Belic J, Jones DT, Tschida B, Moriarity B (2015). Somatic CRISPR/Cas9-mediated tumour suppressor disruption enables versatile brain tumour modelling. Nat Commun.

[3] Alexandrov LB, Ju YS, Haase K, Van Loo P, Martincorena I, Nik-Zainal S, Totoki Y, Fujimoto A, Nakagawa H, Shibata T (2016). Mutational signatures associated with tobacco smoking in human cancer. Science.

[4] Braganza MZ, Rajaraman P, Park Y, Inskip PD, Freedman ND, Hollenbeck AR, Berrington de Gonzalez A, Kitahara CM (2014). Cigarette smoking, alcohol intake, and risk of glioma in the NIH-AARP Diet and Health Study. Br J Cancer.

[5] Ahn S, Han KD, Park YM, Bae JM, Kim SU, Jeun SS, Yang SH (2020). Cigarette Smoking Is Associated with Increased Risk of Malignant Gliomas: A Nationwide Population-Based Cohort Study. Cancers.

[6] Li HX, Peng XX, Zong Q, Zhang K, Wang MX, Liu YZ, Han GL (2016). Cigarette smoking and risk of adult glioma: a meta-analysis of 24 observational studies involving more than 2.3 million individuals. Onco Targets Ther.

