# Supplementary material for: Tobacco smoking associates with NF1 mutations exacerbating survival outcomes in gliomas
Source: Biomark Res. 2022 Nov 9;10:78. doi: 10.1186/s40364-022-00430-z (PMC9648001; doi:10.1186/s40364-022-00430-z)
Supplement: Supplementary file 1 — Additional file 1. [file 40364_2022_430_MOESM1_ESM.docx]

**Supplementary Material for: Tobacco smoking associates with *NF1* mutations exacerbating survival outcomes in gliomas**

Xi Li; Han Yan; Jun Wu^*^; Longbo Zhang^*^

**Method of targeted sequencing:**

Genomic DNA libraries were constructed via Illumina TruSeq DNA Library Preparation Kit. Libraries were hybridized to custom-designed biotinylated oligonucleotide probes (Roche NimbleGen) covering ~1.1 Mbp of 1021 genes. DNA sequencing was carried out with GeneSeq2000 with 100-bp paired-end reads. After collection of the sequencing data, clean reads were aligned to the reference human genome (hg19) using bwa mem. Somatic single nucleotide variants (SNVs) and small insertions and deletions (InDel) were identified using MuTect and GATK software respectively by filtering out germline mutations. Statistical analyses and graphic visualization were performed using R (version 4.1.1, Maftools and Survminer packages) and GraphPad Prism 8. Statistical significance was determined using Fisher’s Exact test, and Log-Rank test, with *P* < 0.05 for significance for all comparisons.

**1021-gene panel**

**Somatic mutations:**

All 4847 exons of 312 genes

| ABL1 | ACVR1B | AKT1 | AKT2 | AKT3 | ALK | APC | AR | ARAF | ARID1A |
| --- | --- | --- | --- | --- | --- | --- | --- | --- | --- |
| ARID1B | ARID2 | ASXL1 | ATM | ATR | ATRX | AURKA | AURKB | AXIN1 | AXIN2 |
| AXL | B2M | BAP1 | BARD1 | BCL2 | BCL2L1 | BCOR | BLM | BMPR1A | BRAF |
| BRCA1 | BRCA2 | BRD4 | BRIP1 | BTK | CARD11 | CASP8 | CBFB | CBL | CCND1 |
| CCND2 | CCND3 | CCNE1 | CD274 | CDC73 | CDH1 | CDK12 | CDK4 | CDK6 | CDK8 |
| CDKN1A | CDKN1B | CDKN2A | CDKN2B | CDKN2C | CEBPA | CHEK1 | CHEK2 | CIC | CREBBP |
| CRKL | CSF1R | CTCF | CTNNA1 | CTNNB1 | CUL3 | CYLD | DAXX | DDR1 | DDR2 |
| DICER1 | DNMT3A | DOT1L | EGFR | EIF1AX | C11orf30 | EP300 | EPAS1 | EPCAM | EPHA2 |
| EPHA3 | EPHA5 | EPHB1 | EPHB6 | ERBB2 | ERBB3 | ERBB4 | ERCC1 | ERCC3 | ERCC4 |
| ERCC5 | ERG | ERRFI1 | ESR1 | EXT1 | EXT2 | EZH2 | FAM123B | FAM175A | FANCA |
| FANCC | FANCD2 | FANCE | FANCF | FANCG | FANCL | FANCM | FAS | FAT1 | FAT2 |
| FBXW7 | FGF19 | FGF3 | FGF4 | FGFR1 | FGFR2 | FGFR3 | FGFR4 | FH | FLCN |
| FLT1 | FLT3 | FLT4 | FOXA1 | FOXL2 | FOXP1 | FUBP1 | GALNT12 | GATA3 | GNA11 |
| GNAQ | GNAS | GRIN2A | GRM3 | HDAC1 | HGF | HNF1A | HOXB13 | HRAS | IDH1 |
| IDH2 | IFNG | IFNGR1 | IGF1R | IKBKE | IKZF1 | IL7R | INPP4B | IRF2 | IRS2 |
| JAK1 | JAK2 | JAK3 | JUN | KDM5A | KDM5C | KDM6A | KDR | KEAP1 | KIT |
| KRAS | LRP1B | MAF | MAP2K1 | MAP2K2 | MAP2K4 | MAP3K1 | MAPK1 | MAX | MCL1 |
| MDM2 | MDM4 | MED12 | MEF2B | MEN1 | MET | MITF | MLH1 | MLH3 | MLL |
| MLL2 | MLL3 | MPL | MRE11A | MS4A1 | MSH2 | MSH3 | MSH6 | MST1R | MTOR |
| MUTYH | MYC | MYCL1 | MYCN | MYD88 | NBN | NCOR1 | NF1 | NF2 | NFE2L2 |
| NFKBIA | NKX2-1 | NOTCH1 | NOTCH2 | NOTCH3 | NPM1 | NRAS | NSD1 | NTHL1 | NTRK1 |
| NTRK2 | NTRK3 | PALB2 | PARK2 | PARP1 | PAX5 | PBRM1 | PCK1 | PDCD1 | PDCD1LG2 |
| PDGFRA | PDGFRB | PDK1 | PIK3CA | PIK3CB | PIK3CG | PIK3R1 | PIK3R2 | PMS1 | PMS2 |
| POLD1 | POLE | POT1 | PPP2R1A | PRDM1 | PRKAR1A | PTCH1 | PTCH2 | PTEN | PTPN11 |
| PTPRD | RAC1 | RAD50 | RAD51 | RAD51B | RAD51C | RAD51D | RAD52 | RAD54L | RAF1 |
| RARA | RB1 | RBM10 | RECQL | RECQL4 | RET | RHOA | RICTOR | RINT1 | RNF43 |
| ROS1 | RPTOR | RUNX1 | SDHA | SDHAF2 | SDHB | SDHC | SDHD | SERPINB3 | SERPINB4 |
| SETD2 | SF3B1 | SLX4 | SMAD2 | SMAD3 | SMAD4 | SMARCA4 | SMARCB1 | SMO | SOCS1 |
| SOX2 | SOX9 | SPOP | SRC | STAG2 | STAT3 | STK11 | SUFU | SYK | TBX3 |
| TCF7L2 | TERC | TET2 | TGFBR2 | TMEM127 | TMPRSS2 | TNFAIP3 | TNFRSF14 | TOP1 | TOP2A |
| TP53 | TSC1 | TSC2 | TSHR | U2AF1 | VEGFA | VHL | WRN | WT1 | XPO1 |
| XRCC2 | ZMAT3 | - | - | - | - | - | - | - | - |

Introns, promoters, and fusion breakpoints of 38 genes

| ALK | BCL2L11 | BRAF | BRCA1 | BRD4 | CD74 | EGFR | EML4 | ERG | ETV6 |
| --- | --- | --- | --- | --- | --- | --- | --- | --- | --- |
| EZR | FGFR1 | FGFR2 | FGFR3 | KIF5B | KIT | MAML2 | MET | MSH2 | MYC |
| MYCL1 | NCOA4 | NOTCH2 | NTRK1 | NTRK2 | NTRK3 | PDGFRA | RAF1 | RET | ROS1 |
| RSPO2 | SDC4 | SLC34A2 | TERT | TFE3 | TMPRSS2 | TPM3 | PMS2 | - | - |

1778 coding regions in 709 genes

| ABCA13 | ABCB1 | ABCC1 | ABCC11 | ABCC2 | ABCG2 | ABL2 | ACACA | ACIN1 | ACTB |
| --- | --- | --- | --- | --- | --- | --- | --- | --- | --- |
| ACTG1 | ACTG2 | ACVR2A | ACVRL1 | ADAM29 | ADAMTS5 | ADCY1 | AFF1 | AFF2 | AFF3 |
| AHNAK | AKAP9 | ALB | AMOT | ANGPT1 | ANK3 | ANKRD11 | ANKRD30A | ANKRD30B | APEX1 |
| APOBEC3B | ARAP3 | ARFGEF1 | ARFGEF2 | ARHGAP29 | ARHGAP35 | ARID4B | ARID5B | ARNT | ASCL4 |
| ASH1L | ASMTL | ASPM | ASTN1 | ASXL2 | ATIC | ATP11B | ATP12A | ATP1A1 | ATP2B3 |
| BAZ2B | BBC3 | BBS9 | BCAS1 | BCL10 | BCL11A | BCL11B | BCL2A1 | BCL2L11 | BCL3 |
| BCL6 | BCL9 | BCORL1 | BCR | BIRC3 | BMPR2 | BNC2 | BPTF | BRD2 | BRD3 |
| BRSK1 | BRWD1 | BTLA | BUB1 | C15orf23 | C15orf55 | C1QA | C1S | C3orf70 | C7orf53 |
| C8orf34 | CACNA1E | CADM2 | CALR | CAMTA1 | CASP1 | CASQ2 | CBLB | CBR1 | CBR3 |
| CCDC168 | CCNA1 | CCNB3 | CCT3 | CCT5 | CCT6B | CD22 | CD33 | CD5L | CD74 |
| CDA | CDH11 | CDH18 | CDH23 | CDK13 | CHD1 | CHD1L | CHD4 | CHD6 | CHD8 |
| CHD9 | CHFR | CHI3L1 | CHN1 | CIITA | CLDN18 | CLP1 | CLSPN | CLTC | CNOT3 |
| CNOT4 | CNTN1 | CNTN5 | CNTNAP1 | CNTNAP5 | COL1A1 | COL2A1 | COL5A1 | COL5A2 | COL5A3 |
| COPS2 | CPS1 | CRIPAK | CRLF2 | CRNKL1 | CRTC1 | CSF1 | CSF3R | CSMD1 | CSMD3 |
| CSNK1A1 | CSNK1G3 | CTLA4 | CTNNA2 | CTNND1 | CUX1 | CXCR4 | CYBA | CYP19A1 | CYP1A1 |
| CYP1B1 | CYP2A13 | CYP2C8 | CYP2D6 | CYP3A4 | CYP3A5 | DCC | DDX3X | DDX5 | DEK |
| DHX35 | DHX9 | DIAPH1 | DIS3L2 | DLC1 | DMD | DNAH6 | DNAJB1 | DNM2 | DNMT1 |
| DNMT3B | DOCK2 | DOCK7 | DPYD | DRGX | DTX1 | DUSP22 | DYSF | E2F3 | EBF1 |
| ECT2L | EED | EEF1A1 | EGFL7 | EGR3 | EIF2AK3 | EIF2C3 | EIF3A | EIF4A2 | EIF4G3 |
| ELAC2 | ELF1 | ELF3 | ELMO1 | ELN | EME2 | EMID2 | EML4 | EPC1 | EPHA1 |
| EPHA4 | EPHA7 | EPHB2 | EPHB4 | EPOR | EPPK1 | EPS15 | ERBB2IP | ERCC2 | ESR2 |
| ETS1 | ETV1 | ETV5 | ETV6 | EWSR1 | EZR | F8 | FAM131B | FAM135B | FAM157B |
| FAM46C | FAM5C | FAP | FASLG | FAT3 | FAT4 | FCGR1A | FCGR2A | FCGR2B | FCGR3A |
| FCRL4 | FGF10 | FGF12 | FGF14 | FGF23 | FGF6 | FLG | FLI1 | FLNC | FMN2 |
| FN1 | FNDC4 | FOXA2 | FOXO1 | FOXO3 | FOXQ1 | FRMPD4 | FUS | FXR1 | FYN |
| FZD1 | G3BP1 | G3BP2 | GAB2 | GABRA6 | GATA1 | GATA2 | GFRAL | GIGYF1 | GKN2 |
| GLB1L3 | GLI1 | GLI2 | GLI3 | GMPS | GNA13 | GNG2 | GPC3 | GPR124 | GPS2 |
| GPX1 | GRB7 | GSK3B | GSTM5 | GSTP1 | GUSB | H3F3A | H3F3B | H3F3C | HCLS1 |
| HCN1 | HDAC4 | HDAC9 | HECW1 | HEY1 | HIST1H1C | HIST1H1D | HIST1H1E | HIST1H2AC | HIST1H2AG |
| HIST1H2AL | HIST1H2AM | HIST1H2BC | HIST1H2BD | HIST1H2BJ | HIST1H2BK | HIST1H2BO | HIST1H3B | HIST1H3C | HIST1H3D |
| HIST1H3F | HIST1H3G | HIST1H3H | HIST1H3I | HIST1H4I | HIST3H3 | HLA-A | HLA-B | HLA-C | HLF |
| HMCN1 | HNF1B | HNRPDL | HOXA11 | HOXA13 | HOXA3 | HOXA9 | HOXC13 | HOXD11 | HOXD13 |
| HSD3B1 | HSP90AA1 | HSP90AB1 | HSPA8 | HSPD1 | HSPH1 | ICK | ICOSLG | ID3 | IFITM3 |
| IGF1 | IGF2 | IGF2R | IGLL5 | IKZF2 | IKZF3 | IL10 | IL1RAPL1 | IL21R | IL6 |
| IL6ST | IMPG1 | ING1 | INHBA | INPP4A | INPPL1 | INSR | IRF4 | IRF6 | IRS1 |
| ITGB3 | ITK | ITSN1 | JARID2 | KALRN | KAT6A | KAT6B | KCNJ5 | KCNQ2 | KDM2B |
| KEL | KIF5B | KLF4 | KLHL6 | KLK1 | KRTAP5-5 | L3MBTL1 | LAMA2 | LATS1 | LATS2 |
| LCP1 | LEF1 | LGALS8 | LIFR | LPHN2 | LPP | LRP2 | LRP4 | LRP5 | LRP6 |
| LRRC7 | LRRK2 | LYN | LZTS1 | MACF1 | MAD1L1 | MAGI2 | MAML2 | MAML3 | MAP3K13 |
| MAPK3 | MCC | MCM3 | MDC1 | MECOM | MEF2C | MGA | MIB1 | MIOS | MKL1 |
| MLL4 | MLLT3 | MMP11 | MMP2 | MN1 | MNDA | MNX1 | MSH4 | MSN | MSR1 |
| MTHFR | MTRR | MUC5B | MYH11 | MYH14 | MYH9 | MYO3A | MYOD1 | NAP1L1 | NAV3 |
| NCAM2 | NCF2 | NCF4 | NCK1 | NCOA3 | NCOA4 | NCOR2 | NCSTN | NDUFA13 | NFATC4 |
| NFE2L3 | NKX3-1 | NLRC3 | NOD1 | NOS3 | NOTCH4 | NQO1 | NR1I2 | NR2F2 | NR4A2 |
| NRG1 | NRP2 | NRXN1 | NTM | NUMA1 | NUP107 | NUP210 | NUP93 | NUP98 | OBSCN |
| OGDH | OMD | OPCML | OR11G2 | OR2T4 | OR4A15 | OR4C6 | OR5L2 | OR6F1 | P2RY8 |
| P4HB | PABPC1 | PABPC3 | PAG1 | PAK1 | PAK3 | PASK | PAX3 | PAX7 | PC |
| PCDH18 | PCSK6 | PCSK7 | PDCD11 | PDE4DIP | PDGFB | PDILT | PER1 | PGR | PHF1 |
| PHF6 | PIK3C2A | PIK3C2B | PIK3C2G | PIK3C3 | PIM1 | PKD1L2 | PKHD1 | PLAG1 | PLCB1 |
| PLCG1 | PLCG2 | PLK1 | PLXNA1 | PLXNB2 | PNRC1 | POLQ | POM121 | POM121L12 | POU2AF1 |
| PPM1D | PPP1R17 | PPP6C | PRDM16 | PREX2 | PRF1 | PRKAA1 | PRKCB | PRKCI | PRKDC |
| PRRX1 | PRX | PSG2 | PSIP1 | PSMB1 | PSMB5 | PTGS1 | PTGS2 | PTPN13 | PTPN2 |
| PTPRB | PTPRK | PTPRO | PTPRS | PTPRT | PTPRU | RAB35 | RAC2 | RAD21 | RAD54B |
| RANBP2 | RASA1 | RASGRP1 | RBL1 | REL | RELN | RFC1 | RGS3 | RHEB | RHOH |
| RHOT1 | RIT1 | RNASEL | ROBO1 | ROBO2 | ROBO3 | ROCK1 | RPGR | RPS6KB1 | RPS6KB2 |
| RSPO2 | RSPO3 | RUNX1T1 | RUNX2 | RXRA | RYR1 | RYR2 | SBDS | SCUBE2 | SDC4 |
| SEC31A | SEMA3A | SEMA3E | SEMA6A | SERPINA7 | SETBP1 | SETDB1 | SF1 | SF3A1 | SFPQ |
| SGCZ | SGK1 | SH2B3 | SH2D1A | SH3PXD2A | SHH | SI | SIN3A | SLC16A1 | SLC1A2 |
| SLC22A16 | SLC22A18 | SLC22A2 | SLC22A3 | SLC34A2 | SLCO1B3 | SLIT1 | SLIT2 | SMARCD1 | SMARCE1 |
| SMC1A | SMC1B | SNCAIP | SNTG1 | SNX29 | SOD2 | SOS1 | SOX10 | SOX17 | SPEN |
| SPRR3 | SPSB4 | SPTA1 | SRD5A2 | SRGAP1 | SRGAP3 | SRSF2 | SRSF7 | STAG1 | STAT1 |
| SUCLG1 | SUCLG2 | SULT1A1 | SUZ12 | SVEP1 | SYNCRIP | SYNE1 | TAF1 | TAF15 | TAF1L |
| TAL1 | TBL1XR1 | TBX15 | TBX22 | TCEB1 | TCF12 | TCF3 | TCF4 | TCL1A | TEC |
| TENM3 | TERT | TET1 | TFDP1 | TFDP2 | TFE3 | TGFBR1 | THBS2 | TJP1 | TLE1 |
| TLL2 | TLR4 | TLX3 | TMEM132D | TNFSF11 | TNN | TP53BP1 | TP63 | TP73 | TPM3 |
| TPR | TRAF2 | TRAF7 | TRIM24 | TRIM58 | TRIO | TRPC5 | TRRAP | TSHZ2 | TSHZ3 |
| TTF1 | TUBA3C | TUBB3 | TUSC3 | TXNIP | TYMS | TYR | UBE2D2 | UBR5 | UGT1A1 |
| UMPS | UPF3B | USH2A | USP6 | USP8 | VEZF1 | VIM | VTCN1 | WASF3 | WDR90 |
| WDTC1 | WHSC1 | WHSC1L1 | WIPF1 | WNK1 | WNT5A | WSCD2 | WWOX | WWP1 | WWP2 |
| XIAP | XPC | XRCC1 | XRCC3 | YAP1 | YY1AP1 | ZBTB16 | ZC3H11A | ZFHX3 | ZFP36L1 |
| ZFP36L2 | ZFPM2 | ZIC3 | ZNF217 | ZNF384 | ZNF521 | ZNF638 | ZNF750 | ZNF804B | - |

**Germline mutations:**

All exons of 91 genes

| APC | ATM | ATR | ATRX | AXIN2 | BAP1 | BARD1 | BLM | BMPR1A | BRCA1 |
| --- | --- | --- | --- | --- | --- | --- | --- | --- | --- |
| BRCA2 | BRIP1 | CDC73 | CDH1 | CDK12 | CDK4 | CDKN1B | CDKN2A | CHEK1 | CHEK2 |
| CTNNA1 | EPCAM | ERCC1 | EXT1 | EXT2 | FAM175A | FANCA | FANCC | FANCD2 | FANCE |
| FANCF | FANCG | FANCL | FANCM | FH | FLCN | GALNT12 | HOXB13 | KIT | MAX |
| MEN1 | MET | MLH1 | MLH3 | MRE11A | MSH2 | MSH3 | MSH6 | MUTYH | NBN |
| NF1 | NF2 | NTHL1 | NTRK1 | PALB2 | PDGFRA | PMS2 | POLD1 | POLE | PTEN |
| RAD50 | RAD51 | RAD51B | RAD51C | RAD51D | RAD52 | RAD54L | RB1 | RECQL | RECQL4 |
| RET | RINT1 | RNF43 | SDHA | SDHAF2 | SDHB | SDHC | SDHD | SLX4 | SMAD4 |
| SMARCA4 | SMARCB1 | STK11 | TMEM127 | TP53 | TSC1 | TSC2 | VHL | WRN | WT1 |
| XRCC2 | - | - | - | - | - | - | - | - | - |
